# Epigenetic control in thyroid cancer: mechanisms and clinical perspective

**DOI:** 10.1038/s41420-025-02688-2

**Published:** 2025-08-17

**Authors:** Jiahui Zhang, Shengkai Zheng, Ruiwang Xie, Junsi Zhang, Xiangjin Chen, Sunwang Xu

**Affiliations:** 1 Department of Thyroid and Breast Surgery, the First Affiliated Hospital, Fujian Medical University, Fuzhou, 350005 China; 2 Department of Thyroid and Breast Surgery, National Regional Medical Center, Binhai Campus of the First Affiliated Hospital, Fujian Medical University, Fuzhou, 350212 China; 3 Fujian Provincial Key Laboratory of Precision Medicine for Cancer, Fuzhou, 350005 China

**Keywords:** Cancer, Cell biology

## Abstract

Epigenetic regulation plays a key role in the progression, proliferation, and dedifferentiation of thyroid cancer. Epigenetic control occurs at multiple levels, including DNA methylation, RNA modification, histone modification, chromatin remodeling, and chromatin accessibility. Genetic alterations in chromatin regulators are commonly observed in thyroid cancer, which includes papillary thyroid carcinoma (PTC), medullary thyroid carcinoma (MTC), anaplastic thyroid carcinoma (ATC), and follicular thyroid carcinoma (FTC). These cancers exhibit distinct characteristics in terms of genetics, biology, and clinical presentation. Therefore, we review the disease biology driven by changes in chromatin pathways in thyroid cancer. Specifically, we summarize examples of epigenetic dysregulation at each level, with mechanisms involving alterations in enzymes regulating DNA methylation, RNA modification and posttranslational modifications of histones, as well as the loss or fusion of subunits involved in chromatin remodeling and chromatin accessibility. Finally, on the basis of clinical applications, we review the current and potential future approaches for thyroid cancer treatment.

## FACTS

- Thyroid cancer has garnered considerable research attention as the most prevalent endocrine malignancy.
- Select ATC and metastatic thyroid cancer cells are resistant to diverse therapies, including radiotherapy, chemotherapy, multikinase inhibitors, and immune checkpoint inhibitors.
- The oncogenesis of thyroid cancer involves multidimensional regulatory networks and includes dysregulated epigenetic modifications, epithelial-to-mesenchymal transition (EMT), tumor cell metabolic reprogramming, and aberrantly activated signaling pathways.

## QUESTIONS

- How does multilevel epigenetic regulation synergistically drive tumor dedifferentiation and progression in thyroid cancer?
- How do genetic alterations in chromatin regulators establish subtype-specific epigenetic dysregulation patterns across thyroid cancer histotypes?
- Can the targeting of key nodes in chromatin pathways overcome therapy resistance in thyroid cancer? What fundamental challenges impede their clinical translation?

## Introduction

Thyroid cancer is the most common malignant endocrine tumor, leading to 40 000 deaths each year [1]. Most thyroid cancer cases involve follicular cells, and the disease can be divided into different types, including PTC, FTC, and ATC, while a small number of cases are derived from follicular parafollicular cells of MTC [2]. Most early-stage thyroid cancers can be cured through conventional methods such as surgery, radioactive iodine therapy, and thyroid stimulating hormone (TSH) suppression. However, the treatment of locally advanced or metastatic thyroid cancers remains challenging, which negatively impacts disease-specific survival rates. The challenge primarily arises because thyroid cancer cells often exhibit resistance to treatments such as radiotherapy, chemotherapy, multikinase inhibitors, and immune checkpoint inhibitors [3]. The rapid development of targeted biological therapies, particularly multikinase inhibitors, over the past two decades has shown promising efficacy and safety in clinical trials and international studies. However, these treatments still face issues with primary or acquired resistance, which hinders their long-term effectiveness [4].

**Fig. 1:**
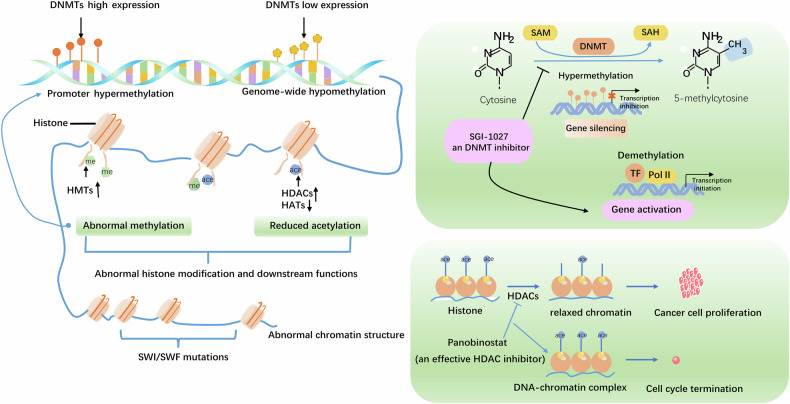
Schematic diagram of the three levels of epigenetic regulation driving the transcriptional program of thyroid cancer. **1** DNA methylation—Promoter hypermethylation or global hypomethylation modulates gene expression. **2** Histone modifications—mutations in histone PTMs and enzyme-mediated methylation/acetylation alter gene regulation. **3** Chromatin remodeling—SWI/SNF complex dysfunction disrupts nucleosome positioning and transcription factor binding.

These challenges have spurred researchers to explore the underlying mechanisms of thyroid cancer, particularly its epigenetic mechanisms [5–7]. Epigenetic treatment strategies have shown early promise. For example, the U.S. Food and Drug Administration has approved epigenetic drugs such as ivosidenib and enasidenib for the treatment of acute myeloid leukemia, both of which lower 2-hydroxyglutarate levels and promote myeloid cell differentiation. Additionally, tazemetostat, a zeste homolog 2 (EZH2) inhibitor that promotes cell differentiation, has been approved for the treatment of follicular lymphoma [8]. Given the promising efficacy of epigenetic drugs in other cancers and the reversibility of epigenetic modifications, many researchers are hopeful that these drugs could provide new therapeutic options for thyroid cancer. Substantial progress has been made in this field in recent years. Epigenetic regulation of gene expression occurs through a multitiered mechanism without altering DNA sequences [9]. First, at the DNA methylation level, cytosine methylation, specifically promoter hypermethylation or global hypomethylation, directly modulates gene expression. Second, with respect to histone modifications, nucleosomes, the functional units of chromatin composed of four core histones, are regulated by methylation/acetylation enzyme complexes, with mutations in key amino acids disrupting posttranslational modifications (PTMs) and driving thyroid cancer progression. Finally, in chromatin remodeling, complexes such as SWI/SNF regulate nucleosome positioning to influence DNA accessibility for transcription factor binding, and their dysfunction leads to chromatin structural abnormalities that promote thyroid carcinogenesis (Fig. 1). This review systematically elucidates the molecular mechanisms, clinical significance, and research limitations of these epigenetic modifications in thyroid cancer, providing cutting-edge insights and future perspectives for this emerging field.

## Chromatin accessibility

Chromatin is composed of repeating nucleosome units, which are composed of a histone octamer core and approximately 147 base pairs of wrapped DNA, joined by regions of linker DNA. Its variable composition, structure, and dynamics can regulate genomic processes, influencing the binding capacity of transcription factors and their ability to carry out normal functions such as DNA repair and replication. A key aspect of chromatin regulation lies in the ability of factors to acquire and maintain physical access to DNA, commonly referred to as DNA or chromatin accessibility [10].

ATC is one of the most lethal cancers and is characterized by dedifferentiation and a high degree of metastasis [11]. It exhibits stem cell-like properties and high proliferative potential. Due to dedifferentiation, ATC does not exhibit normal thyroid function and thyroid differentiation markers, making chemotherapy and radiotherapy ineffective. For example, ATC cells do not express the thyroid-stimulating hormone receptor (TSHR), leading to resistance to TSH suppression therapy, while the loss of the sodium iodide symporter (NIS) results in resistance to radioactive iodine therapy [12]. Chromatin remodeling and chromatin accessibility are crucial for tumor initiation and progression, but their role in promoting ATC dedifferentiation is not well understood. Hematological and neurological expressed 1 (HN1) promotes dedifferentiation in ATC cells [13]. HN1, through its interaction with STMN1, drives ATC metastasis by destabilizing microtubules. Additionally, HN1 exhibits significant nuclear localization in ATC cells, although its exact function in the nucleus remains unclear [13].

Further investigation in an independent thyroid cancer cohort revealed a negative correlation between HN1 and CTCF, with HN1 inhibition enhancing CTCF expression in ATC cells [13]. Moreover, ATAC-seq and ChIP-seq analyses confirmed that CTCF regulates genes related to thyroid development by influencing chromatin accessibility [13]. HN1 suppresses CTCF transcriptional activation by recruiting histone deacetylase 2 to deacetylate H3K27 at the CTCF promoter, thereby regulating the chromatin accessibility of thyroid differentiation genes. These findings indicate that HN1 plays a crucial role in modulating the chromatin accessibility of thyroid differentiation genes during the dedifferentiation of ATC, thus promoting the dedifferentiation of ATC cells [13].

Chromatin accessibility serves as a marker for active enhancers, which are cis-regulatory elements responsible for regulating gene expression [14]. Typically, chromatin accessibility is measured in conjunction with RNA abundance to construct a gene expression regulatory landscape, although proteomic data are not included in this analysis. Although RNA is often used as a surrogate marker for protein expression, numerous studies have indicated a significant discrepancy between RNA and protein levels (~correlation = 0.3) [15, 16]. Compared with upstream distal enhancers, proximal enhancer regions were found to be more actively involved in driving changes in protein abundance. These regions may play a key role in the development of malignancy and could be the most reliable predictors of cancer gene expression phenotypes [17]. Proteins regulated by these enhancers could influence cancer phenotypes, making them promising targets for diagnostic and therapeutic strategies. Furthermore, the researchers hypothesized that in rapidly proliferating cells, protein levels are more closely linked to gene expression and chromatin accessibility because the regulation of genes by downstream proximal enhancers is less affected [17].

## Chromatin remodeling complexes

Chromatin remodeling involves the ATP-dependent repositioning or reassembly of nucleosomes, which can influence the dynamic competition between histones and transcription factors for cis-regulatory sequences in gene promoters, and is critical for cell differentiation and tumorigenesis [18, 19].

### SWI/SNF

Mutations in the subunits of the SWI/SNF chromatin remodeling complex are commonly found in various human cancers, including advanced thyroid cancer. These mutations can lead to a range of cancer-related changes, such as abnormal cell proliferation, lineage differentiation, and metabolic alterations [20, 21]. Playing a crucial role in gene expression regulation, the SWI/SNF complex is composed of 12 to 15 subunits and uses the energy from ATP hydrolysis to mobilize nucleosomes and remodel chromatin. This complex is often targeted to enhancers located distal from gene transcription start sites, many of which are closely related to developmental processes and lineage specificity [22]. The SWI/SNF complex is typically divided into three subclasses: BRG1/BRM-associated factor (BAF), polybromo-associated factor (PBAF), and noncanonical BAF (ncBAF) [23]. These three subclasses share some core subunits, such as SMARCC1/2 and SMARCD1/2/3, but each also contains unique subunits. For example, the BAF complex contains ARID1A or ARID1B and ARID2, while the PBAF complex contains PBRM1, and the ncBAF complex includes GLTSCR1 and BRD9. Mutations in the subunit genes of the SWI/SNF complex are recurrently found in ~20% of cancer cases [23, 24]. In addition, the SWI/SNF complex plays an important role in tumor initiation and progression [25], particularly in certain cancer types [26], where it interacts with and antagonizes the activity of polycomb repressive complex 2 (PRC2). Loss of function of SWI/SNF is thought to promote tumorigenesis by activating PRC2 and upregulating stem cell-associated programs [26].

A recent study revealed that the specific loss of Arid1a, Arid2, or Smarcb1 (SWI/SNF complex) in thyroid cancer with the BRAFV600E mutation in mice promotes disease progression and results in reduced survival rates [27]. This process is associated with changes in chromatin accessibility and lineage-specific differentiation. Compared with normal thyroid cells, BRAFV600E-mutant mice with PTC exhibit decreased expression of lineage transcription factors and reduced accessibility to their target DNA binding sites, leading to impaired expression of thyroid differentiation genes and reduced radioactive iodine uptake. This can be partially alleviated by mitogen-activated protein kinase (MAPK) inhibitors. However, the loss of a single SWI/SNF subunit in BRAF-mutant tumors induces a repressive chromatin state, resulting in the loss of chromatin accessibility of thyroid lineage-specific genes; this state cannot be reversed by blocking the MAPK pathway, making the tumors unresponsive to redifferentiation effects [27].

When activated by SETMAR, the SWI/SNF complex promotes differentiation in thyroid cancer [28]. Importantly, SMARCA4 and SMARCA2 are two critical catalytic subunits within the SWI/SNF complex that are mutually exclusive, yet they share a high degree of homology [29]. Additionally, the paralog dependency model has been used to explain the SWI/SNF complex, indicating that SMARCA2 and SMARCA4 can compensate for one another’s loss [30].

Mutations in SWI/SNF result in increased dependency on EZH2, a methyltransferase in the PRC2 complex that is responsible for catalyzing histone methylation [31, 32]. The enhancer of EZH2 is overexpressed in ATC cells, where it inhibits paired box gene 8 (PAX-8) transcription and thereby blocks thyroid cell differentiation. While the mechanisms by which SWI/SNF mutations affect tumor progression and interact with other oncogenic factors are not fully understood, increasing evidence suggests that these effects are lineage and context dependent [33]. A study [27] revealed that the loss of different SWI/SNF subunits leads to distinct chromatin and transcriptional profiles, promoting thyroid dedifferentiation and tumor progression. Notably, the loss of the SWI/SNF complex has also been associated with resistance to RAF/MEK inhibitor-based redifferentiation therapy in mouse models and some clinical trial patients [27].

### Reprogramming cancer differentiation states

Considerable progress has been made in the development of therapeutic interventions that target different states of cancer, particularly in myeloid malignancies. A prototypical example is acute promyelocytic leukemia, which is caused by the PML–RARα fusion gene, a product of the recombination between the promyelocytic leukemia (PML) gene and the retinoic acid receptor-α (RARα) gene. This fusion protein blocks myeloid differentiation at the promyelocyte stage in the bone marrow [34], and treatment with all-trans retinoic acid and arsenic trioxide leads to remission [35]. A similar mechanism applies to thyroid cancer, where the differentiation status of the tumor is a key determinant of its response to treatment. PTC is the most common form and is characterized by relatively simple genetic mutations, which primarily activate signaling through the MAPK pathway [36], such as BRAFV600E (60%), RAS (15%), and gene fusions involving BRAF, RET, NTRK, and ALK (12%) [37]. In contrast, next-generation sequencing studies in poorly differentiated thyroid cancer (PDTC) and ATC reveal a more complex mutational landscape, with an increasing mutational burden [38, 39] and a progressive accumulation of TERT promoter and TP53 mutations [39, 40]. A particularly notable feature is the development of mutations in genes encoding epigenetic modifiers, most notably the subunits of the SWI/SNF (BAF and PBAF) chromatin remodeling complexes [39]. The functional relevance of this discovery is supported by in vivo Sleeping Beauty transposon mutagenesis screening, which revealed that the disruption of chromatin modifiers, including SWI/SNF subunits, significantly cooperates with oncogenic HRAS during the progression of PDTC [41].

## Histone modification

The ability of a gene to be transcribed is dependent on the conformation and accessibility of chromatin and is also controlled by histone activity at the posttranslational level. Histone modification refers to the PTMs of amino acids at the N-terminus of histones, including acetylation, methylation, and ubiquitination [42]. These PTMs of histones can alter chromatin structure, for example, by changing it from tightly packed heterochromatin to loosely packed euchromatin, thereby regulating gene expression.

Acetylation and deacetylation of lysine residues in histones have been widely reported to provide balance in controlling gene transcription in an epigenetic level. The enzymes responsible for these acetylation and deacetylation processes are HATs and HDACs. HATs catalyze the acetylation of histones, which leads to chromatin relaxation and enhances gene expression. In contrast, HDACs remove acetyl groups, causing chromatin compaction and the repression of gene expression [43]. The acetylation of histone lysine residues prevents the interaction between histones and DNA by removing the negative charge on histones. Unlike methylation, histone acetylation is associated with increased gene transcription. Historically, histone acetylation has been extensively studied, particularly in cancer. For example, it appears to play a role in the early stages of thyroid cancer. Immunohistochemical analyses revealed higher levels of H3K18ac and H3K9-K14ac in PTC and FTC tissues than in control tissues, whereas only H3K9-K14ac was detected in ATC tissues [44]. This observation suggests that the lack of H3K18Ac expression in ATC may play an oncogenic role during the dedifferentiation of this form of thyroid cancer.

The lysine and arginine residues at the N-terminal tails of histones serve as sites for methylation, which results in the monomethylation, dimethylation, or trimethylation of histones [45]. A recent study revealed that histone H3 lysine 4 and H3 lysine 9 demethylase KDM1A are frequently overexpressed in PTC tissues and cell lines. Reduced expression of KDM1A suppresses the migration and invasion of PTC cells both in vitro and in vivo [46]. Furthermore, histone methyltransferases such as KMT2D and KMT5A seem to play pivotal roles in the epigenetic modifications associated with thyroid cancer [47, 48]. Histone methylation modifiers, such as EZH2, have been shown to be overexpressed in ATC cells, thereby inhibiting the transcription of PAX-8 [49] and further promoting the dedifferentiation of ATC.

Furthermore, histone deacetylation can also regulate gene expression through epigenetic mechanisms, thus promoting the dedifferentiation of thyroid cancer cells. In thyroid cancer, genes regulated by deacetylation include “differentiation genes”, such as NIS, thyroglobulin, TTF-1, and thyroid peroxidase [50]. An effective HDAC inhibitor, panobinostat, exhibits cytotoxic effects on ATC cell lines both in vitro and in vivo, inducing cell cycle arrest and apoptosis in mouse xenograft models while blocking tumor growth [51]. Importantly, this treatment also induces SLC5A5 mRNA expression, leading to a corresponding increase in NIS. Additionally, the effects of two other HDAC inhibitors, suberoylanilide hydroxamic acid (SAHA) and trichostatin A, have been confirmed [52]. The potential to reinduce NIS expression in advanced and RAI-refractory differentiated thyroid tumors has prompted numerous clinical trials using various HDAC inhibitors, such as SAHA [53], vorinostat [54], romidepsin [55], valproic acid [56] and depsipeptide [57]. However, regardless of the subtype of thyroid cancer, cell lines with BRAFV600E or HRAS mutations respond more poorly to HDAC inhibitor treatment than those with other genetic alterations. The combination of HDAC inhibitors with MAPK or PI3K/Akt inhibitors has been shown to enhance antitumor effects in thyroid cancer cell lines [51]. Moreover, although preclinical evidence suggests that histone deacetylase inhibitors may promote the redifferentiation of ATC [58], the clinical impact of this potential treatment strategy has not yet been established, and there are no data available on PDTC.

Bromodomain-containing protein 4 (BRD4), an epigenetic regulator that specifically recognizes and binds to acetylated histones through its bromodomain and then acts as a scaffold protein to recruit the transcription elongation factor b (P-TEFb) complex to facilitate the transcription of acetylated chromatin regions, plays a crucial role in the onset and progression of various diseases, including thyroid cancer [59–61]. Gao et al. examined the expression levels of BRD4 in thyroid tumors and its potential as a therapeutic target. Notably, BRD4 was found to be overexpressed in PTC samples compared with normal tissue, indicating that BRD4 may contribute to the progression of thyroid cancer [59]. In ATC, the Aurora family members A, B, and C are overexpressed and mediate mitotic regulation via the control of histone H3 phosphorylation and chromatin remodeling [62]. Currently, research on histone modifications in MTC is limited. The genes encoding the histone methyltransferases EZH2 and SMYD3 are overexpressed in patients with invasive MTC that has metastasized to the lymph nodes and distant organs, but this overexpression is unrelated to the mutation status of the RET and RAS genes [63].

### METTL3-SETMAR-SMARCA2-TF axis

Most thyroid cancer patients have well-differentiated PTC, but in some cases, the disease may later transform into PDTC or even ATC, which significantly worsens the prognosis [64]. Additionally, PDTC and ATC exhibit increased proliferative potential and display features of EMT [39]. Therefore, revealing the mechanisms controlling the transformation between differentiation and dedifferentiation in thyroid cancer is urgently needed to identify therapeutic targets and approaches for PDTC and ATC.

A recent study revealed the role of the METTL3-SETMAR-SMARCA2-TF axis in the differentiation of thyroid cancer [28]. This study revealed that METTL3-mediated m6A modifications (RNA modifications) maintain SETMAR mRNA stability via the m6A reader IGF2BP3 in well-differentiated thyroid cancer. SETMAR facilitates the dimethylation of H3K36 (H3K36me3) at the SMARCA2 promoter region, promoting SMARCA2 transcription. As a result, SMARCA2 binds to the enhancers of the thyroid differentiation transcription factors (TFs) PAX8 and FOXE1, enhancing their expression by increasing chromatin accessibility. PAX8 and FOXE1 are genes associated with thyroid differentiation. The METTL3-SETMAR-SMARCA2-TF axis unites RNA modifications, histone modifications, chromatin remodeling and accessibility to drive the differentiation of thyroid cancer [28].

SETMAR, a histone methyltransferase containing an N-terminal histone-lysine N-methyltransferase catalytic domain and a C-terminal transposase domain, possesses catalytic activity for methylation at histone H3K4me2, H3K36me2, and H3K27me3 sites, thereby regulating gene transcription [65, 66]. As a downstream target of SETMAR via histone methylation, SMARCA2, a core component of the SWI/SNF complex, catalyzes ATP hydrolysis via its ATPase subunit to forcibly alter the spatial positioning or stability of nucleosomes and supply the energy for chromatin remodeling [67]. Therefore, SETMAR-activated SMARCA2 enhances chromatin accessibility for the active expression of thyroid differentiation transcription factors through chromatin remodeling to regulate thyroid cancer differentiation [28].

In clinical treatment, selumetinib, a MEK inhibitor that inhibits the activity of the MEK1 and MEK2 enzymes, thereby indirectly blocking the transmission of the MAPK/ERK signaling pathway, has been used in radioactive iodine-resistant thyroid cancer patients and has shown significant clinical effects [68, 69]. Additionally, the authors reported that, compared with wild-type SETMAR, overexpression of SETMAR with a methyltransferase-deficient mutation did not enhance the MAPK-induced redifferentiation effect [28]. However, the METTL3-14-WTAP activator can enhance the redifferentiation effect of MAPK inhibitors. Therefore, combining METTL3-14-WTAP activators with MAPK inhibitors can be used for ATC, providing a new therapeutic direction [28].

## RNA modifications

RNA modifications play a key role in regulating processes such as cell proliferation, differentiation, invasion, migration, stemness, metabolism, and drug resistance, thus influencing tumorigenesis either by promoting or inhibiting it. Among the various RNA modifications, N6-methyladenosine (m6A), 5-methylcytosine (m5C), and N7-methylguanosine (m7G) have been linked to the development of tumors [70].

### m5C modification and ATC

m5C modification refers to the methylation of the fifth carbon atom of cytosine in RNA, which occurs in various RNA molecules, including tRNA, rRNA, mRNA, and noncoding RNA (ncRNA). m5C modification plays a key role in maintaining RNA stability and regulating protein synthesis and translation [71]. Methylation of m5C is regulated by ‘writers’, ‘erasers’ and ‘readers’. Writers refer to enzymes that catalyze the addition of methylations, such as members of the DNMT and NSUN families; erasers are enzymes that remove these modifications, such as members of the TET family; and readers specifically recognize methylation marks, such as Aly/REF nuclear export factor and Y-box binding protein 1, thereby converting epigenetic signals into biological functions [70].

A previous study revealed that the m5C methyltransferase NSUN2, which catalyzes tRNA m5C modification, can stabilize the secondary structure of tRNA in ATC, facilitating rapid amino acid transport, particularly leucine transport [72]. This stable tRNA supports oncogenic translation programs for genes, including c-Myc, BCL2, RAB31, JUNB, and TRAF2, significantly increasing their efficiency. This effect promotes codon-dependent oncogenic translation and leads to malignant dedifferentiation and reprogramming of cancer protein translation [72].

Knockout of NSUN2 was shown to suppress tumor formation, proliferation, and metastasis while increasing chemosensitivity and disrupting the c-Myc-NSUN2 feedback loop in ATC [72]. This knockout significantly reduces m5C modification at the C48 position of tRNA Leu-CAA, although some tRNAs show increased m5C levels due to compensatory DNMT2 activity, maintaining tRNA modification homeostasis [73]. Furthermore, the knockout of NSUN2 also affects the expression of tRNA Leu-CAA, indicating that unmethylated tRNA is less stable and more prone to fragmentation [74]. The differences in translation efficiency between tRNA Leu-CAG and Leu-CAA may lead to codon bias in ATC cells [72]. Specifically, to maintain malignant translational reprogramming, tumor cells require Leu-CAA, which is a more stable tRNA than Leu-CAG. This finding is consistent with the role of tRNA Leu-CAG in the translation of tumor suppressors [75].

### m6A modifications in PTC and ATC

**Fig. 2:**
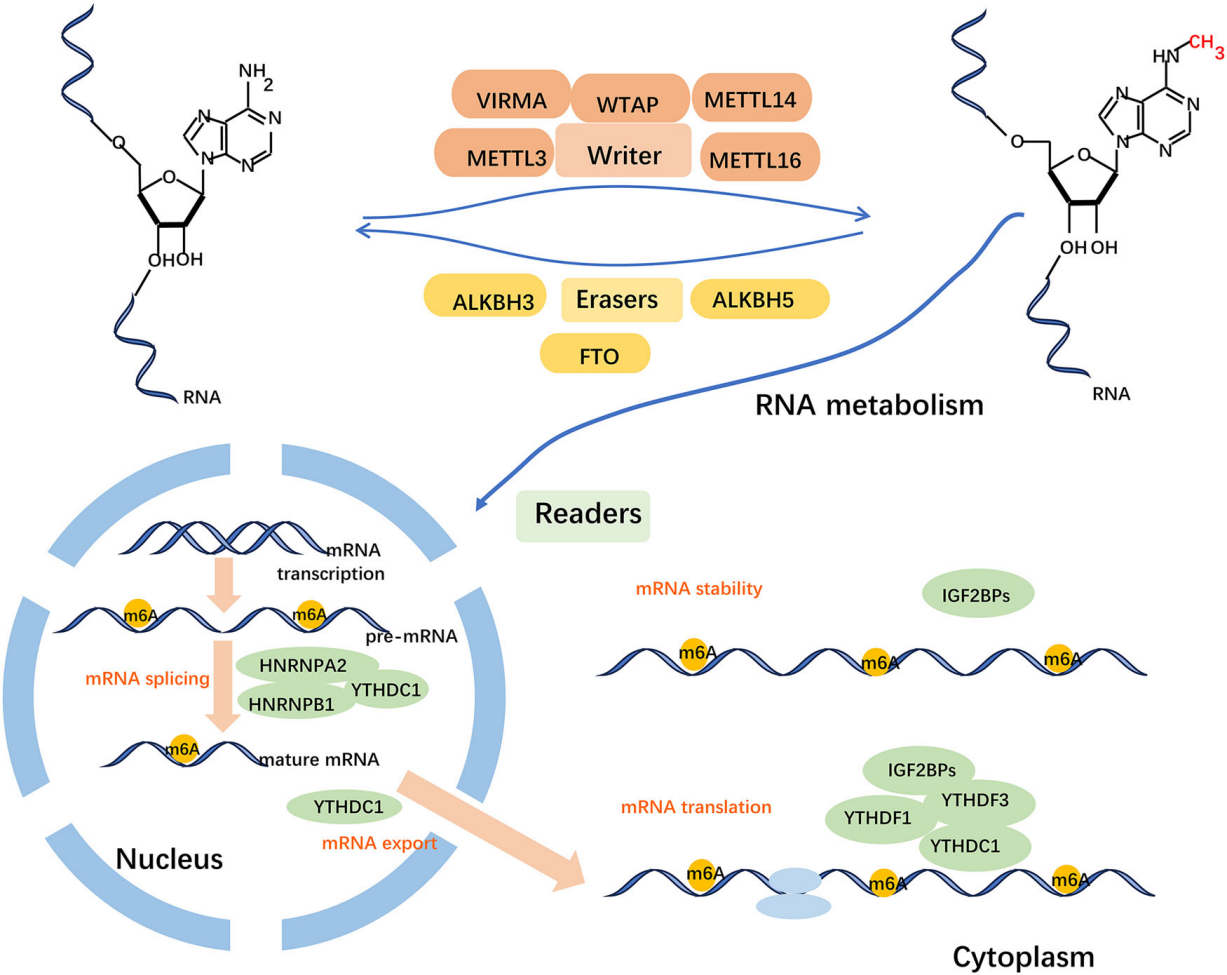
m6A modification mechanism. m6A RNA methylation is regulated by “writers,” “erasers,” and “readers.” The writers mainly include the METTL3-METTL14 complex, WTAP, VIRMA, and METTL16, which are methyltransferases that promote m6A methylation. The erasers include FTO, ALKBH5, and ALKBH3, which induce m6A demethylation and are demethylases. The readers include YTHDF1-3, YTHDC1, IGF2BPs, and HNRNPs (HNRNPC1, HNRNPG, HNRNPA2, and HNRNPB1), which are involved in RNA splicing, processing, nuclear export, decay, translation, and stability.

N_6_-Methyladenosine, the most abundant posttranscriptional modification in RNAs, including mRNAs and ncRNAs, regulates multiple steps in RNA processing, including splicing, export, translation, and degradation, thereby controlling target gene expression (Fig. 2) [76, 77]. This process is primarily catalyzed by three types of enzymes, also known as m6A regulators, including methylases (‘writers’), demethylases (‘erasers’) and m6A-binding proteins (‘readers’), making m6A modification dynamic and reversible [78]. Increasing evidence shows that m6A modification is an important cancer biological process that regulates carcinogenesis, tumor progression, and drug resistance [79, 80]. RNA m6A modification refers to the addition of a methyl group at the N6 position of adenosine and tends to occur in DRACH (*D* = G/A/U, *R* = G/A, and *H* = A/C/U) [81]. The most familiar motif is GGACU, which is located in 3′-untranslated regions (UTRs), coding sequences, stop codon regions, and 5′-UTRs [82]. The common m6A writers are the METTL3-14 complex [83], Wilms’ tumor 1-associating protein (WTAP) [84], and KIAA1429 (VIRMA) [85]. The common m6A erasers are obesity-associated protein (FTO) [86], AlkB homolog 5 (ALKBH5) [87], and ALKBH3 [88]. The common m6A readers are YTHDF1-3, YTHDC1-2 [89, 90], and HNRNP [91].

#### FTO, PTC, and ATC

The m6A demethylase FTO can inhibit glycolysis and progression in PTC [86]. Apolipoprotein E (APOE) is a target gene of FTO-mediated m6A RNA modification, with the RNA-binding protein involved in this process being IGF2BP2. Moreover, APOE promotes tumor growth and proliferation in PTC through glycolysis, and analysis has shown that the FTO/APOE axis inhibits PTC glycolysis by modulating the IL-6/JAK2/STAT3 signaling pathway [86]. These findings reveal a new molecular mechanism by which m6A modification regulates the progression of PTC, providing new insights for the development of effective treatment strategies for PTC.

Previous studies have reported that FTO has different roles in various cancers, exhibiting both tumor-promoting and tumor-suppressing effects, suggesting that the role of FTO in cancer may be dependent on its microenvironmental context. For example, in breast tumors, FTO promotes tumor progression by inhibiting BNIP3 [88], whereas another study reported that FTO suppresses metastasis by demethylating the mRNA of metastasis-associated protein 1 in colorectal cancer [92]. However, in ovarian cancer, FTO acts as a tumor suppressor by regulating cyclic AMP signaling, which is involved in stemness and tumor initiation [90].

Another study indicated that decreased FTO enhances the stability of cadherin-12 (CDH12) mRNA through m6A modification mediated by IGF2BP2 to promote CDH12 expression [93]. As a key subtype of the N-cadherin family, CDH12 enhances the invasion and metastasis of B-CPAP, CAL-62, and 8305 C cells through the EMT pathway, thereby promoting invasion and metastasis in PTC and ATC. These findings suggest that CDH12 is a key target gene of FTO [93]. EMT is a pivotal biological process that enables tumor cells to acquire metastatic potential. Through the downregulation of epithelial markers such as E-cadherin and the upregulation of mesenchymal markers, including N-cadherin, vimentin and MMP9, tumor cells gain stem cell-like properties, significantly enhancing their invasive and metastatic capabilities [94]. Therefore, this study reveals a new molecular mechanism regulated by m6A modification in PTC and ATC, suggesting that FTO, IGF2BP2, and CDH12 may be potential therapeutic targets for PTC and ATC patients with significant invasiveness or distant metastasis, providing new directions for the development of effective therapeutic strategies for thyroid cancer.

In addition, SLC7A11 was confirmed as a downstream target of FTO-demethylated m6A modification in PTC [95]. SLC7A11, an amino acid antiporter, facilitates the uptake of extracellular cysteine in exchange for glutamate. A decrease in SLC7A11 expression disrupts the balance between glutathione synthesis and cysteine uptake, leading to the inhibition of glutathione peroxidase 4 and the activation of ferroptosis [96]. Thus, FTO-mediated demethylation of m6A on SLC6A11 mRNA to activate ferroptosis might be a tumor suppressor in PTC progression.

#### ALKBH5 and TC

ALKBH5, another RNA m6A demethylase, also functions as a tumor suppressor in thyroid cancer cells by decreasing the expression of GPX4 and SLC7A11 to induce ferroptosis [97]. In addition, ALKBH5 overexpression downregulates TIAM1 expression by eliminating the m6A modification of TIAM1 mRNA, suppresses thyroid cancer cell proliferation and promotes ferroptosis by regulating the Nrf2/HO-1 pathway [97]. TIAM1 was first discovered in mouse T-lymphoma cells and identified as a metastasis-associated gene [98]. Previous studies have indicated that TIAM1 serves as an oncogene that promotes thyroid cancer metastasis and EMT through the Wnt/β-catenin pathway [99]. Overall, the m6A demethylase ALKBH5 induces ferroptosis to inhibit thyroid cancer progression in an m6A-demethylation-dependent manner.

#### METTL3, ATC, and PTC

Methyltransferase-like 3 (METTL3), an RNA m6A methylase that is silenced in thyroid cancer cells, promotes an immunosuppressive phenotype through CD70 demethylation [100]. CD70 is a TNF family immune checkpoint molecule that regulates T-cell signaling and proliferation. Its overexpression in cancer cells facilitates immune evasion [101]. Moreover, in the PTC and ATC, M2EVs can suppress METTL3 expression, leading to the demethylation and stabilization of CD70 mRNA, resulting in increased levels of the CD70 protein. This, in turn, increases the abundance of immunosuppressive Tregs and terminally exhausted T cells and chemotactically recruits M2 macrophages, thereby forming an immunosuppressive positive feedback loop [100]. The anti-CD70 monoclonal antibody cusatuzumab can disrupt the positive feedback loop and reverse resistance to anti-PD-1 therapy, providing a new strategy for the treatment of PTC and ATC [100].

Another study revealed that the downstream targets of METTL3 m6A modification are c-Rel and RelA [102]. In some cases of PTC, low METTL3 expression was observed, which was associated with the progression of thyroid cancer and poor prognosis. These findings suggest that METTL3 may have a tumor-suppressive role. By promoting METTL3 expression, the levels of m6A modification and YTHDF2 are increased, thereby destabilizing c-Rel mRNA and reducing interleukin-8 (IL-8) secretion. This alteration changed the infiltration of tumor-associated neutrophils and inactivated the NF-kB pathway. The findings of this study provide new insights into the epigenetic molecular mechanisms of PTC and suggest that the METTL3/c-Rel/IL-8 axis could be an important therapeutic target for treating PTC [102].

PAX8, a member of the paired box family and a critical cytoskeletal component, regulates NIS transcription and is essential for thyroid development and maintaining the differentiated state of mature thyroid cells [103]. NIS dysfunction is caused by aberrant activation of the MAPK and PI3K pathways, impairing the iodine-uptake capacity of thyroid follicular cells [18, 19]. The METTL3/PAX8/YTHDC1 axis plays a key role in suppressing PTC progression and dedifferentiation [104]. Mechanistically, low METTL3 expression reduces PAX8 levels via YTHDC1-mediated m6A modification, and decreased PAX8 further downregulates NIS [104]. Additionally, miR-493-5p suppresses METTL3 by directly targeting its mRNA [104]. Overall, miR-493-5p and METTL3 could serve as biomarkers for the diagnosis and treatment of PTC, providing new therapeutic strategies for PTC.

#### METTL16 and thyroid cancer

METTL16, an m6A writer, was found to be highly expressed in thyroid cancer and to promote the proliferation, migration, and invasion of thyroid cancer [105]. The same study identified SAMD11 as a target gene of METTL16, with METTL16 regulating SAMD11 expression through m6A modification, thus playing a crucial role in thyroid cancer. This study provided new potential therapeutic targets for thyroid cancer and lay a theoretical foundation for the study of RNA methylation in thyroid cancer [105].

### m7G modification and thyroid cancer

m7G modifications are found in various types of RNA, such as mRNA, tRNA, and lncRNA, and in the 5’ cap of eukaryotic mRNA [106]. The m7G cap is crucial for RNA stability, nuclear export, splicing, and translation initiation. This cap structure is essential for the efficient binding of mRNA to the ribosome, thereby promoting protein synthesis [107]. In tRNA, m7G modifications can influence tRNA folding, stability, and function, affecting the efficiency and accuracy of protein translation [108]. Increasing evidence suggests that abnormal levels of m7G can promote tumorigenesis by regulating the expression of various oncogenes and tumor suppressor genes. In mammals, the most well-studied regulator of m7G modification is methyltransferase-like 1 (METTL1), which, along with its cofactor WD repeat domain 4 (WDR4), performs m7G modification in tRNA, miRNA, and mRNA. The RNA guanine-7 methyltransferase and its cofactor RAM also participate in m7G modification of the 5’ cap of mRNA by activating small proteins [109].

Zhou et al. [110]. established a prognostic differential expression model (PDEm7G-lncRNA) for thyroid cancer using five m7G-related lncRNAs (DOCK9-DT, DPP4-DT, TMEM105, SMG7-AS1, and HMGA2-AS1); they found that the model significantly aids in early prediction and clinical intervention for high-risk and poor-prognosis patients. Patients with higher scores in this model had a worse prognosis. This research reveals a new pattern of mRNA translation regulation mediated by m7G-modified tRNA and highlights the critical role of m7G modifications in cancer progression [110].

Another study revealed that METTL1-mediated tRNA m7G modification plays a critical oncogenic role in PTC [111]. METTL1 was significantly upregulated in PTC tissues and was associated with poor prognosis. Mechanistically, METTL1 regulates the abundance of specific m7G-modified tRNAs, thereby influencing the codon-specific translation of TNF-α mRNA. Clinical analysis confirmed positive correlations among METTL1, WDR4, and TNF-α expression. This research reveals a novel mechanism of RNA epigenetic modification in PTC and suggests that targeting the METTL1-m7G-TNF-α pathway may represent a promising therapeutic strategy for PTC [111]. Research on the role of m7G in thyroid cancer is still in its early stages, and more studies are needed to explore the biological mechanisms by which m7G modifications impact thyroid cancer.

### Noncoding RNA and PTC

Noncoding RNAs are a class of RNA sequences that do not encode proteins but act as regulators of gene and protein expression, enhancing our understanding of the diverse roles and functions of RNA. Noncoding RNAs include miRNAs, long noncoding RNAs (lncRNAs) and circular RNAs (circRNAs) [112]. CircRNAs are a class of stable noncoding RNAs with a circular structure that are abundant in mammalian cells [113]. CircRNAs play key regulatory roles in gene expression networks at the transcriptional, epigenetic, and posttranscriptional levels [114]. Recent studies have reported associations between certain circRNAs and the oncogenic process of PTC [115]. hsa_circ_0101050 was found to promote the invasion, migration, and proliferation of PTC cells through m6A modification mediated by zinc finger CCCH-type containing 13 (ZC3H13). The same study revealed that ZC3H13 overexpression inhibits PTC cell viability, migration, and invasion. These findings provide new potential targets for the development of effective treatment strategies for PTC [115].

Another study revealed that the downregulation of the m6A demethylase ALKBH5 can promote glycolysis in PTC cells via the circRNA nuclear receptor-interacting protein 1 (circNRIP1) [116]. Additionally, circNRIP1 acts as a sponge for oncogenic miR-541-5p and miR-3064-5p, consequently upregulating the expression of pyruvate kinase M2, a key glycolytic enzyme, thereby facilitating the progression of PTC. Therefore, circNRIP1 could serve as a prognostic biomarker and therapeutic target for PTC [116].

Recent research has revealed an oncogenic mechanism mediated by Circ_0067934 in thyroid cancer pathogenesis. Circ_0067934 functions as a “sponge” for miR-545-3p, inhibiting its activity in thyroid cancer cells and thereby upregulating the ferroptosis-negative regulator SLC7A11, which in turn mitigates ferroptosis in these cells [117]. These findings suggest that Circ_0067934 may serve as a potential therapeutic target for thyroid cancer through the regulation of ferroptosis [117].

## DNA methylation

DNA methylation is a reversible covalent modification carried out by a group of DNA methyltransferases (DNMTs), which add methyl groups to the DNA, with common examples being DNMT1, DNMT3A, and DNMT3B [112]. These enzymes primarily methylate the cytosine in CpG dinucleotides at the carbon-5 position, generating 5mC. Most CpG sites in the human genome are methylated, with the exception of CpG-rich regions, which are typically located near transcription start sites and are associated with promoter regions, known as CpG islands. DNA methylation of CpG islands suppresses gene expression by inhibiting the binding of transcription factors and/or the recruitment of chromatin-modifying enzymes [112, 118]. In cancer, DNA methylation has two mechanisms. One is the site-specific hypermethylation of regulatory elements such as promoters, leading to the silencing of tumor suppressor genes or genes important for cellular functions such as DNA repair and apoptosis. The other is the hypomethylation of all DNA, which can affect extensive domains of the genome and promote genomic instability [119].

Some studies [120] have analyzed the methylation status of specific genes in PDTC and ATC and reported that the alteration rates are generally greater than those in differentiated thyroid cancer. With respect to global methylation, regardless of genotype, 93.8% of patients with PDTC and ATC (a total of 16 patients) exhibited global hypomethylation, whereas only 3.6% of patients with well-differentiated thyroid cancer and 42.4% of patients with metastatic well-differentiated thyroid cancer presented hypomethylation [120]. For example, in one study [121], high RASSF1 methylation of the CpG island in the RASSF1A promoter was found in all patients with PDTC and in 78% of ATC cases, but it was relatively low in differentiated thyroid cancer [121]. Other tumor suppressor genes, such as p16 (INK4A), TSHR, MGMT, DAPK, ESR1, ESR2, RARβ, PTEN, CD26, SLC5A8, and UCHL1, are always methylated in ATC, in contrast to differentiated thyroid cancer [122]. Various pan-cancer studies have shown that PTC has one of the lowest levels of organization in DNA methylation, whether in the overall DNA or in gene promoters [37, 123]. The number of hypomethylation events is greater than that in normal thyroid tissue [119, 124]. In fact, the BRAFV600E mutation is more closely associated with the classic subtype of PTC and is also related to an increase in DNA hypomethylation, whereas RAS mutations are more commonly observed in FTC and PTC and are associated with hypermethylation. Another interesting phenomenon is that the frequency of methylation changes in ATC is 10 times greater than that in PTC, indicating the overall DNA hypomethylation and hypermethylation of CpG islands [119, 125]. The characteristics of FTC include a very high methylation or hypomethylation ratio [119, 124, 126]. For example, the PTEN promoter is highly methylated in thyroid cancer but is more commonly expressed in FTC. If PTEN is not expressed, PI3K may be activated, which is a key step in the survival and dedifferentiation of follicular thyroid cells [127].

A study [128] revealed that DNMT3B-induced methylation of FAM111B promotes glycolysis in PTC. Estrogen can recruit DNMT3B to the FAM111B promoter, thereby promoting the progression of thyroid cancer. The DNMT inhibitor SGI-1027 has been shown to inhibit glycolysis, proliferation, migration, and invasion in PTC cells. Therefore, the use of SGI-1027 may be an effective therapeutic strategy against PTC growth and metastasis [128].

Hypermethylation of the CpG island in the ID3 promoter leads to downregulation of ID3 in PTC cells, and this suppressed ID3 expression is strongly correlated with lymph node metastasis and poor postoperative prognosis in PTC patients [129]. The downregulation of ID3 promotes PTC cell invasion and metastasis by enhancing E47-mediated EMT. Consequently, targeted therapies aimed at reversing ID3 expression or blocking E47 DNA binding could improve the prognosis of PTC patients. For example, the DNA methylation inhibitor Aza has been shown to restore ID3 transcription in PTC cells [129]. However, the tumor-suppressive role of ID3 in tumor initiation and other related malignant behaviors requires further exploration.

Another study [130] revealed the critical role of serine hydroxymethyltransferase-2 (SHMT2) in PTC metastasis, showing that SHMT2 is elevated in PTC and is associated with a poor prognosis. Its overexpression promotes PTC metastasis both in vitro and in vivo. Mechanistically, SHMT2 catalyzes serine metabolism to generate S-adenosylmethionine (SAM), which is utilized for DNA methylation (CpG islands) in the PTEN promoter, thereby suppressing PTEN and activating the AKT signaling pathway [130].

## Clinical applications and the role of epigenetics in thyroid cancer

**Fig. 3:**
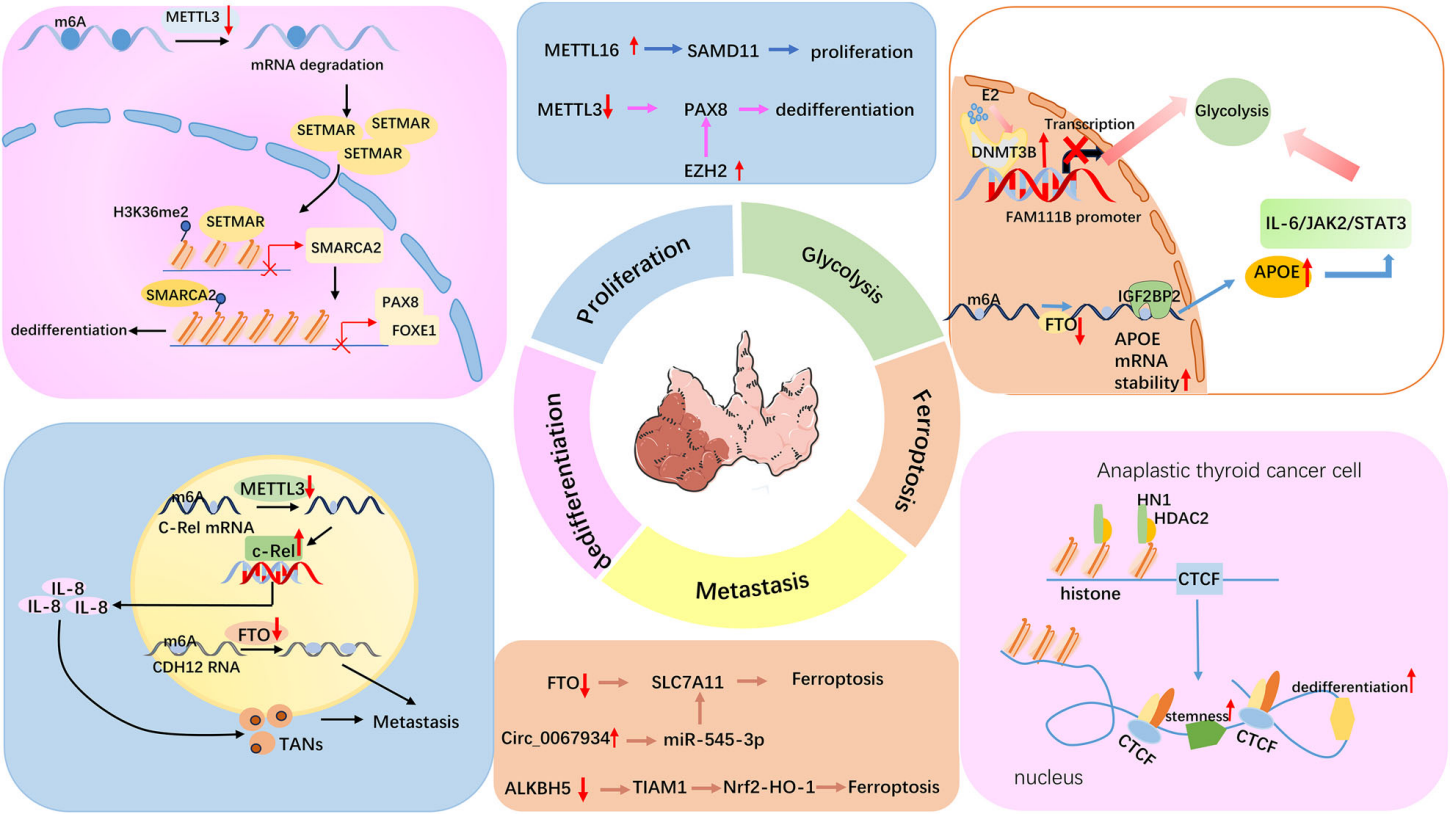
Key Differentially expressed proteins regulated by epigenetic mechanisms in thyroid cancer. Oncogenic regulators, including METTL16, DNMT3B, EZH2, Circ_0067934, and HN1, are highly expressed in thyroid cancer, promoting cell proliferation, dedifferentiation, and glycolysis, while inhibiting ferroptosis. On the other hand, tumor suppressor regulators, such as METTL3, FTO, and ALKBH5, are downregulated in thyroid cancer, suppressing re-differentiation, metastasis, and glycolysis, thereby inhibiting tumor progression and promoting ferroptosis. ↑indicates upregulation, ↓indicates downregulation.

In recent years, epigenetic research has had a substantial impact on the clinical treatment of thyroid cancer (Fig. 3). For example, in the area of DNA methylation, SGI-1027, a nonnucleoside DNMT inhibitor that inhibits DNMT1-, DNMT3A- and DNMT3B-catalyzed DNA hypermethylation [131, 132], has been shown to inhibit glycolysis, proliferation, migration, and invasion of PTC cells, demonstrating its potential for PTC therapy [128]. In the field of histone modification, panobinostat, an HDAC inhibitor that belongs to the cinnamic hydroxamic acid class of compounds that inhibits HDAC1, 3–6 at nanomolar concentrations [133, 134], has exhibited cytotoxic effects by inducing cell cycle arrest and apoptosis in ATC cell lines both in vitro and in vivo [51], but its clinical efficacy remains limited. Although the results of clinical trials using histone deacetylase inhibitors (HDACis) alone for thyroid cancer treatment have been underwhelming, many in vitro studies have further explored the potential of combination therapies, which have shown promising results [135, 136]. Typically, these studies combine two epigenetic inhibitors with different mechanisms, such as HDACis combined with DNMTis [135], or pair one epigenetic inhibitor with another type of inhibitor, such as HDACis combined with tyrosine kinase inhibitors (TKIs), to disrupt the signal transduction pathways of protein kinases [136, 137]. Although the exact mechanisms remain unclear, these combinations appear to be more effective than single-drug treatments are, warranting further investigations in in vivo studies and clinical trials.

In the genetic context of thyroid cancer, identifying genetic events that drive downstream epigenetic changes offers new opportunities for targeted therapies. For example, elevated NSUN2 expression in ATC cells enhances the translation of key transcription factors and antiapoptotic genes, promoting tumor progression and drug resistance [72]. This finding opens new avenues for addressing malignant transformation and resistance. In the context of RNA modifications, FTO, IGF2BP2, and CDH12 may serve as promising therapeutic targets for aggressive or metastatic PTC and ATC [93]. Additionally, ALKBH5 induces ferroptosis through the m6A-TIAM1-Nrf2/HO-1 axis, thereby inhibiting thyroid cancer progression, suggesting that ALKBH5 could be a potential target for both the diagnosis and treatment of thyroid cancer [97].

Several molecular pathways, such as the METTL3/c-Rel/IL-8 axis [102], ZC3H13/hsa_circ_0101050 axis [115], and ALKBH5/circNRIP1/PKM2 axis [116], are closely associated with thyroid cancer progression and poor prognosis. Furthermore, the METTL3‒PAX8‒YTHDC1 axis is linked to thyroid cancer dedifferentiation and progression, positioning it as a potential target for PTC treatment. Moreover, miR-493-5p can target and suppress METTL3, suggesting that both miR-493-5p and METTL3 could serve as diagnostic and therapeutic biomarkers for PTC [104]. Additionally, the anti-CD70 monoclonal antibody cusatuzumab has shown promise in reversing resistance to programmed death-1 (PD-1) inhibitors [100], providing a novel strategy for treating both PTC and ATC.

With respect to histone modification, the METTL3-SETMAR-SMARCA2-TF axis is associated with thyroid cancer cell differentiation. METTL3-14-WTAP activators can restore SETMAR expression, promoting thyroid cancer differentiation. When combined with MAPK pathway inhibitors, this approach could be beneficial for treating ATC [28]. METTL3-14 is found in the nucleus, where it is localized to nuclear speckles, and the splicing regulator WTAP is required for this distinct nuclear localization pattern [138]. The MAPK pathway is known to play a major role in the development of many cancers. The most representative proteins of this pathway and the most important protagonists are RAS, RAF, MEK and ERK. These proteins are involved in various cellular programs, such as differentiation, proliferation and apoptosis [139]. In terms of chromatin accessibility, HN1/CTCF is linked to the dedifferentiation and stem cell-like properties of ATC [13], offering new insights into redifferentiation therapies for ATC.

Overall, targeting epigenetic mechanisms in thyroid cancer treatment holds great promise, although further clinical trials are needed. While considerable progress has been made in targeted therapies for locally advanced or metastatic thyroid cancer, the emergence of resistance has prompted the ongoing exploration of additional effective targets.

## Conclusions

As highlighted in this review, substantial progress has been made in understanding the crucial role of epigenetic dysregulation in various pathological types of thyroid cancer. Notably, there has been some progress in the development of targeted therapies for “oncogenes” associated with proliferation and differentiation in thyroid cancer, including genes involved in epigenetic regulation. For example, low expression of PAX8 inhibits the expression of NIS, leading to the dedifferentiation of thyroid cancer cells [104]. However, despite these advances, traditional precision oncology approaches, which combine METTL3-14-WTAP activators and MAPK inhibitors, have not yet achieved satisfactory results in the treatment of thyroid cancer [28]. As a result, future clinical progress in targeting epigenetic dysregulation in thyroid cancer will rely on more comprehensive clinical genomic testing, which will encompass not only genes related to epigenetic pathways but also novel drugs targeting abnormal epigenetic states, along with precision therapies aimed at specific mechanisms such as DNA methylation, RNA modifications, and histone modifications.
